# An Analysis of Socioeconomic Determinants of the Black–White Disparity in Food Insecurity Rates in the US

**DOI:** 10.3390/foods12112228

**Published:** 2023-06-01

**Authors:** Mya Price, Tia Jeffery

**Affiliations:** College of Agriculture, Urban Sustainability, and Environmental Sciences, University of the District of Columbia, 4200, Connecticut Ave., Washington, DC 20008, USA; tia.jeffery@udc.edu

**Keywords:** food insecurity, racial disparities, socioeconomic status

## Abstract

Previous research has not fully explored socioeconomic factors that influence the Black–White food insecurity disparities at the state and county levels in the United States. The goal of this study was to identify socioeconomic determinants associated with the Black–White food insecurity gap in the US at the state and county levels with rigorous quantitative investigation. The 2019 Map the Meal Gap dataset and multivariate regression analyses were used to identify factors associated with the prevalence of the Black–White disparity in food insecurity rates. Unemployment rate and median income gaps were found to be the strongest predictors of the Black–White disparity in food insecurity and the Black food insecurity rates in both state- and county-level models. Specifically, a 1% increase in Black unemployment rate compared with White unemployment rate was associated with a 0.918% and 0.232% increase in the Black–White disparity in food insecurity on average at the state and county levels, respectively. This study highlights the potential root causes of food insecurity and significant socioeconomic determinants associated with the Black–White food insecurity gap at the state and county levels in the US. Policymakers and program creators should implement action plans to address the income disparities and reduce unemployment rates among Blacks to eradicate this gap and ensure equity in food access between Blacks and Whites.

## 1. Introduction

Food insecurity, known as the limited ability to acquire adequate food, remains an issue of great concern in the US. The estimated healthcare costs of hunger and food insecurity were around USD 160 billion [1], which implies a severe economic burden. According to a recent US Department of Agriculture report, about 11.1% of US households of 37.2 million people, including 11.2 million children, experienced food insecurity in 2018 [2]. The burden of household food insecurity has been disproportionately higher for Black than White adults [3,4,5,6,7]. The distribution of food insecurity remained unequal with Blacks affected at a rate of 21.2%, nearly double the national average of 11.1% [2]. Black adults were more than twice as likely to report that their household did not get enough to eat (19% for Black respondents) as White respondents (7%), according to the Census Bureau’s Current Population Survey in 2020. The gap remained elevated during the COVID-19 pandemic, highlighting the need for more intensive analyses of Black–White disparities in food insecurity in the US. Coupled with the fact that the COVID-19 tragedy has devastatingly threatened food security in the US [8,9,10], understanding the socioeconomic factors associated with increased risk of food insecurity and more severe Black–White disparities in food insecurity in the US is a prerequisite to identifying the determinants of food insecurity in general and the racial/ethnic disparities in food insecurity in particular.

In the literature, food insecurity has been described as economic and social problems of food shortages due to various socioeconomic constraints. Food insecurity is related to unemployment, inflation [6], lack of home ownership [11], changes in income [12,13,14], lack of savings [15], low education [16], tax burden, poor health, and social isolation [17]. According to the Census Bureau’s Household Pulse Survey, 35 million people were either “unemployed” or lived with an unemployed family member. Among these individuals, 23 million adults reported that their household did not get enough to eat in 2020. During the COVID-19 pandemic, 80% reported they that “couldn’t afford to buy more food”. The food insecurity rate was several times larger during the pandemic than the prepandemic rate due to rising unemployment and unfavorable financial and economic factors. Unemployment, a negative income shock, was one of the major causes of food insecurity because it led to an inability to afford food [18]. In addition, poverty and low income were strongly connected to food hardship, especially during the pandemic [8,19].

Limited access to food among Blacks and increasingly severe Black–White disparities in food insecurity in the US may intertwine with risk factors, such as unemployment, poverty, and low incomes. Previous research suggests that the concentration of social and economic disadvantage among African Americans is a significant predictor of their higher rates of food insecurity, and the vulnerability is highest among those characterized by low incomes, renting rather than having house ownership, reliance on employment insurance, unemployment benefits, and social assistance [10,12,13,20,21]. Since African Americans were more likely to live in poverty, face unemployment, and have less wealth and fewer assets than their White counterparts, they were most likely to have higher rates of food insecurity. In the context of the current economic crisis, Black individuals were more likely to experience job losses due to the COVID-19 pandemic, and as a consequence, they were more vulnerable to the economic downturn [22]. Generally, the Black working poor are more likely to be at greater risk for hunger and food insecurity as they lose eligibility for food assistance, receive lower food assistance benefits, or do not enroll in such programs [23].

Nevertheless, socioeconomic risk factors associated with Black–White inequities in food insecurity at the state and county levels have not been fully explored. Prior studies illustrated that states with a high proportion of Blacks tend to have lower wages, higher housing costs and food prices, higher unemployment rates, and regressive tax policies, having a higher burden of food insecurity [24]. According to Feeding America’s research on food insecurity projections, 18 out of the 25 counties that had the highest projected food insecurity rates in 2021 are counties with populations that are majority Black. Specifically, Feeding America’s research projected that 21% of Black individuals (1 in 5 adults) may experience food insecurity in 2021, compared with 11% of White individuals (1 in 9 adults) [22,25]. This implies that Blacks have experienced higher rates of food insecurity than Whites in the US, especially after the COVID-19 pandemic. However, there has been limited analysis of how vulnerability to food insecurity disparities relates to socioeconomic risk factors at the state and county levels with rigorous quantitative investigation. To provide a more robust quantitative analysis on Black–White gaps in projected food insecurity rates and to help inform both policies and programs to mitigate increasingly severe Black–White disparities in food insecurity in the US, this study sought to identify socioeconomic determinants associated with the Black–White food insecurity gap in the United States at the state and county levels.

## 2. Data and Methods

### 2.1. Data

We used cross-sectional data from the Map the Meal Gap (MMG) in 2019. MMG data were developed and sourced from the American Community Survey (ACS), Current Population Survey (CPS), and Bureau of Labor Statistics data [12]. The ACS contains demographic, economic, social, and housing status information from a nationally representative sample. The CPS is a nationally representative survey that contains household information on employment status and occupational parameters (hours worked, income, and other similar factors) conducted by the US Census Bureau for the Bureau of Labor Statistics. The data were structured to a scientifically selected multistage probability-based sample of households, with the sample designed to represent the civilian noninstitutional population of each state (as well as the District of Columbia) and the US as a whole.

The MMG data at the state level were derived from the Core Food Security Model (CFSM) within the CPS. Using the household information in the CPS on food insecurity status and socioeconomic factors, including unemployment, poverty, homeownership, and median income, MMG aggregated the data to the state level and race. CPS survey data were mainly used to assess the relationship between food insecurity and its determinants at the state level. County and district level data on the above variables were drawn from the ACS, except for unemployment data, which were drawn from the BLS. 

The data from MMG, originally sourced from CFSM and the CPS, contained food insecurity rates by race and an established rich set of socioeconomic characteristics, including unemployment, poverty, homeownership, and median income. To assess the prevalence of food insecurity, respondents were asked about situations potentially conducive to food scarcities. The data were based on 15 food insecurity questions of the USDA concerning food shortages in the household, including: (1) “We worried whether our food would run out before we got money to buy more. Was that often, sometimes, or never true for you in the last 12 months?” (2) “We couldn’t afford to eat balanced meals. Was that often, sometimes, or never true for you in the last 12 months?” and (3) “In the last 12 months did you or other adults in your household ever not eat for a whole day because there wasn’t enough money for food? If yes, how often did this happen—almost every month, some months but not every month, or in only 1 or 2 months?”

The food insecurity rates at the county level were estimated using a two-step approach. In the first stage, a multivariate regression was used to estimate state-year-level food insecurity rates using state-level data from 2009 to 2018. Variables in the model included unemployment, non-undergraduate student poverty rate, median income, disability rate, percent of homeowners, percent of Blacks, percent of Hispanics, state, and year fixed effects. These variables were selected based on the literature guidance [9]. In the second stage, the projected food insecurity rates at the county level were obtained from the prediction equation in the first stage with county-level explanatory variables based on 2015–2019 ACS 5-year estimates and 2019 BLS 1-year averages. Overall, our analyses included 50 states and the District of Columbia for the state-level regressions and 1675 counties for the county-level regressions.

### 2.2. Methods 

We used univariate analyses to determine the descriptive statistics for food insecurity rates and socioeconomic determinants among Blacks and Whites at the state and county levels. Furthermore, multivariate linear models were performed to examine socioeconomic factors that are significantly associated with the Black–White gap in food insecurity rates in the US in general and food insecurity rates for African Americans in the US.

We used the student *t*-test to examine whether the means of two populations, Blacks and Whites, were different. The two-independent-samples *t*-test was employed when the Black and White populations were compared on one common variable. In this case, the *t*-tests were performed to analyze whether the differences in average food insecurity rates, average poverty rates, average unemployment rates, average homeownership rates, and average median incomes between Blacks and Whites were statistically significant at the state level and county level, respectively. 

To determine the factors that are significantly related to the relationship between food insecurity and Black–White race, we used multivariate regression models at the state level and county level, respectively. The dependent variable was the Black–White food insecurity gap, which was measured as the difference in food insecurity rates between Blacks and Whites. Socioeconomic predictors of the outcome were differences in unemployment rates, differences in poverty rates, differences in homeownership rates, and differences in median incomes between Blacks and Whites.

To identify socioeconomic factors that are significantly associated with the food insecurity rate among Blacks at the state and county levels, given their social and economic disadvantages and the need for policies to help address the severity of the Black food insecurity rate, multivariate regression models were also conducted at the state level and county level, respectively, with the dependent variable of Black food insecurity rates and the independent variables of Black unemployment rates, Black poverty rates, Black homeownership rates, and Black median incomes.

The regression analyses were performed using Feeding America’s Map the Meal Gap (MMG) 2019 data with 50 states and the District of Columbia and 1675 counties.

Model A

$$Difference\_Food\_insecurity\_rate_{i}\;\;\;\;\;\;\;\;\;\;\;\;\;\;\;\;\;\;\;\;\;\;=\alpha +\beta_{1}Dif\_UR_{i}+\beta_{2}Dif\_PR_{i}+\beta_{3}Dif\_MI_{i}+\beta_{4}Dif\_H_{i}+\varepsilon_{i}$$

where 

Difference_Food_insecurity_rate = Difference in food insecurity rates between Blacks and Whites

$Dif\_UR_{i}=$ Difference in unemployment rates between Blacks and Whites

$Dif\_PR_{i}=$ Difference in poverty rates between Blacks and Whites

$Dif\_MI_{i}=$ Difference in median incomes between Blacks and Whites

$Dif\_H_{i}=$ Difference in homeownership rates between Blacks and Whites

Model B

$$Black\_Food\_insecurity\_rate_{i}=\alpha +\beta_{1}B\_UR_{i}+\beta_{2}B\_PR_{i}+\beta_{3}B\_MI_{i}+\beta_{4}B\_H_{i}+\varepsilon_{i}$$

where

$B\_UR_{i}=$ Black unemployment rate 

$B\_PR_{i}=$ Black poverty rate 

$B\_MI_{i}=$ Black median income 

$B\_H_{i}=$ Black homeownership rate

Statistical analyses were performed using STATA statistical software (version 15.1; StataCorp. 2017, Stata statistical software: release 15, College Station, TX, USA: StataCorp LLC.). 

## 3. Results

### 3.1. Descriptive Results

Based on a nationally representative study sample of 51 states/District of Columbia and 1675 counties as the sampling units from MMG 2019, we found that the overall prevalence of food insecurity was significantly higher for Blacks than Whites at both the state and county levels. The detailed characteristics of the sample are displayed in Table 1 and Table 2. The Black food insecurity rate was nearly 20%, which was more than double the White food insecurity rate of 9.63% at the state level. On average, Blacks had a 14.01% higher poverty rate, 4.79% higher unemployment rate, 35.26% lower homeownership rate, and USD 26,703.27 lower median incomes than Whites at the state level in 2019. Similarly, the Black food insecurity rate was significantly higher than the White food insecurity rate at the county level. The Black food insecurity rate was 22.41%, which was twice the White food insecurity rate of 11.07% at the county level in 2019. This can be attributed to the higher poverty rate, the higher unemployment rate, the lower homeownership rate, and the lower median income for Blacks than Whites. On average, Blacks had a 14.90% higher poverty rate, 5.01% higher unemployment rate, 28.94% lower homeownership rate, and USD 20.90 thousand lower median incomes than Whites at the county level in 2019.

#### Black–White Disparities in Food Insecurity and Socioeconomic Characteristics

We found that Blacks experienced significantly higher levels of food insecurity than Whites at both the state and county levels (*p* < 0.01) from the *t*-test results demonstrated in Table 3 and Table 4. The difference in food insecurity rates between Blacks and Whites is 10.33% and 11.07% on average at the state and county levels in 2019, respectively. In terms of socioeconomic factors, the differences between Blacks and Whites in poverty rate, unemployment rate, homeownership rate, and median incomes at the state and county levels were also statistically significant at the 1% level (*p* < 0.01). 

**Figure 1:**
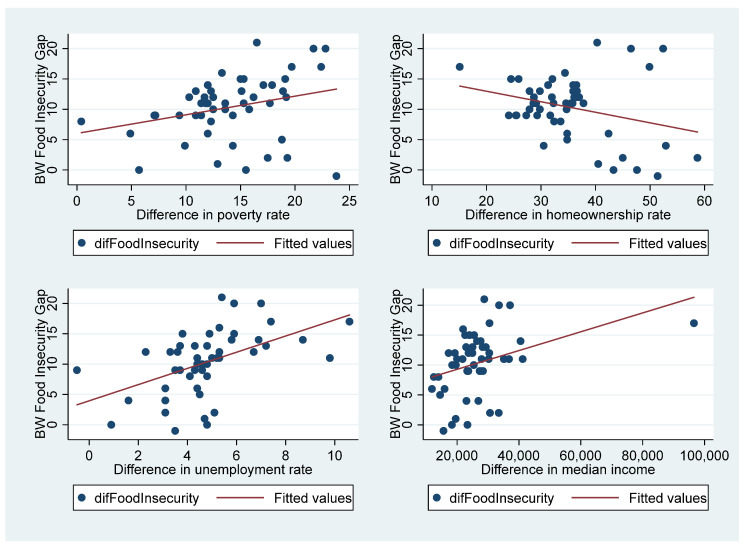
Relationship between Black–White disparity in food insecurity rate and other Black–White difference characteristics at the state level.

As shown in Figure 1, the difference in Black and White food insecurity rates was positively correlated with the gap in unemployment rate, poverty rate, and median income between Blacks and Whites. Other maps in supplemental documents (as shown in Appendix A) show a contrast between the Black food insecurity rate and White food insecurity rate. The maps demonstrated that some states displayed profound differences in food insecurity rates between Blacks and Whites, such as Minnesota, Idaho, Oregon, and Mississippi, which can be explained by the higher unemployment rates and lower median incomes for Blacks than Whites.

### 3.2. Multivariate Results

Table 5 and Table 6 demonstrate the results from Model A, predicting the causes of the Black–White disparity in food insecurity at the state and county levels. Socioeconomic factors, including differences in poverty rate, unemployment rate, homeownership rate, and median income at the state level, were examined in Model A (Table 5). The results suggest that the Black–White gap in unemployment is the significant predictor of Black–White disparity in food insecurity (*p* < 0.05) at the county level. Specifically, Model A’s prediction presents that a 1% increase in the Black unemployment rate compared with the White unemployment rate was associated with a 0.918% increase in the Black food insecurity rate compared with the White food insecurity rate on average at the county level, after controlling for other variables in the model (i.e., Black–White difference in poverty rate, difference in homeownership rate, and difference in median income). 

Results from Model A presented in Table 5, column 2, indicate that the Black–White disparity in food insecurity rates was positively associated with the differences in unemployment rate, poverty rate, median income, and homeownership rate between Blacks and Whites at the county level (*p* < 0.001). The prediction suggests that a 1% increase in the Black unemployment rate compared with the White unemployment rate was associated with a 0.232% increase in the Black food insecurity rate compared with the White food insecurity rate on average at the county level, after controlling for other variables in the model at the county level. 

In addition, we examined socioeconomic factors that influence Black food insecurity rates in 2019. To reduce the gap in food insecurity between Blacks and Whites, we will need to identify Black socioeconomic factors that can reduce this gap. Four multivariate regression models are presented in Table 6 to show how socioeconomic factors are significantly associated with the food insecurity rate among Blacks at the state and county levels. We found that the food insecurity rate among Blacks was associated with unemployment rate, homeownership rate, and median income at the state level. Specifically, Model B in Table 6, column 1, indicates that a 1% increase in the Black unemployment rate was associated with a 0.829% increase in the food insecurity rate among Blacks on average, after controlling for all variables in the model (i.e., poverty rate, median income, and homeownership rate). In addition, a 1000 USD increase in median income among Blacks was associated with a 0.291% lower food insecurity rate among Blacks after controlling for the same variables. Lastly, a 1% increase in the homeownership rate among Blacks was associated with a 0.14% increase in the Black food insecurity rate among Blacks after controlling for the same variables. However, the positive relationship between the Black homeownership rate and the Black food insecurity rate might be inconsistent with our expectation. Similar results were found when the poverty rate was dropped from Model B, columns 2 and 4, due to statistical insignificance. Model B, column 3, suggests that the significant predictors of higher food insecurity rate among Blacks were higher Black unemployment rate, higher Black poverty rate, lower Black median income, and lower Black homeownership rate at the county level (*p* < 0.001). These findings indicate that to reduce the food insecurity rate among Blacks, it is necessary to increase Black median income and homeownership, together with diminishing unemployment rate and poverty rate for Blacks. 

Multicollinearity and heteroscedasticity were diagnosed for these models as well. In this study, multicollinearity was not an issue since no significant correlation among independent variables in the regressions at both the state and county levels was found from the correlation matrix, as shown in Appendix B.

## 4. Discussion

### 4.1. Interpretation of the Results

Our results suggest that difference in unemployment rates between Blacks and Whites is the strongest predictor of the Black–White disparity in food insecurity rates, as it was statistically significant in both state- and county-level models. The average Black food insecurity rate was approximately double the average White food insecurity rate at both the state and county levels. In addition, the average Black unemployment rate was more than double the average White unemployment rate at both the state and county levels. This finding is in light of previous research at the household level that the individuals and families from Black communities are often at risk of suffering from food insecurity [3,4] due to their social and economic disadvantages, such as higher unemployment and loss of jobs [26]. One presumed explanation is that Blacks have been facing long-standing economic hardship as compared with White counterparts [27]. This study contributes further evidence demonstrating that the gap between Blacks and Whites in food insecurity is persistent, is positively correlated with the high Black unemployment rate, and continues to be a chronic issue requiring active policies and programs to address, especially during this pandemic and postpandemic era. 

Furthermore, we found that the Black–White difference in unemployment rates, median income, and poverty rate were statistically significant predictors that can be attributed to the Black–White gap in food insecurity rates at the county level. Based on the model of predicting Black food insecurity rates, and consistent with prior research at the household level, the significant predictors of high levels of food insecurity among African Americans are low median incomes [10,12,13,21], more vulnerability to the economic downturn [18], and high unemployment rates [26]. However, the Black–White disparity in food insecurity rates was not associated with more renting than house ownership [8]. This could be related to the Black working poor being at greater risk for poor financial management, insufficient resources to control for rising mortgage rates, and different market risks [28].

Contrary to Black food insecure adults, White food insecure adults were more likely to report that stores did not have the food they wanted, or the main food access barrier was more likely to be temporary due to supply issues, market shifts, labor shortages, or the COVID-19 pandemic period, where stores experienced certain food shortages [8,19]. However, the burdens of food insecurity among Blacks are more likely to be prolonged due to systemic economic hardship, lower income [10,12,13,21], lower homeownership [8], and higher unemployment [26]. As a solution to mitigate the Black–White disparity of food insecurity, we need to address the root causes, including enacting laws that encourage the creation of new jobs and creating more training programs for Blacks to provide more job opportunities and advancement. These activities can reduce unemployment and improve the financial status of Black individuals in the labor market.

### 4.2. Limitations/Future Research

Since the data were derived from an observational study rather than a natural experiment, these findings illustrate the association or correlation between the Black food insecurity rate and other characteristics as well as the gap in food insecurity rates between Blacks and Whites and its determinants rather than suggesting causality. Another potential limitation of this research could be the omitted variables and endogeneity. If unobservable or unmeasurable factors that affect the food insecurity rate among Blacks or Black-and-White gaps in food insecurity rates exist, such as Black ability, Black-and-White gap in ability or education, and omitted state or county unobserved heterogeneity, we might have some biased estimates. Our analysis of panel data on US food insecurity rates at the state and county levels is not available at this stage. Future studies should expand on data collection to gather panel data of US food insecurity rates across time to control for state fixed effects or county fixed effects. Future research with panel data will address the issue of unobserved heterogeneity at the state or county levels, especially rural vs. urban communities and regional variations of the Mid-Atlantic, Pacific, Midwestern, and Southern areas of the US.

Moreover, it is crucial that future research consider risk factors that are eligible for extensive systematic reviews (education, age, race/ethnicity, and gender). For example, behavioral risk factors and risk factors mapped to the food availability dimension of food security require further investigation to better assess human behavior and environmental factors linked to food availability and barriers that impact Black communities in the United States.

Based on the findings of this paper, further research around the socioeconomic determinants of food insecurity, especially low-income households and systemic racism, should consider distinguishing further by race to determine other socio-characteristics tied to the barriers to food security.

## 5. Conclusions and Policy Implications

The aim of this study was to fill in the gap in the literature by examining the relationship between county-and-state-level socioeconomic determinants and the Black–White gap in food insecurity in the United States. This study examined the difference in food insecurity rates between Blacks and Whites in the US using 2019 data at the state and county levels with 50 states and the District of Columbia, as well as nearly 1700 counties from the Map the Meal Gap (MMG) dataset. Multivariate regression analysis was conducted to estimate the relationship of the difference in projected food insecurity rates between Blacks and Whites based on actual 2009 to 2018 data reported by the sample, including socioeconomic factors, such as differences in unemployment rates, median income, poverty rates, and homeownership rates. In summary, these results suggest that the difference in unemployment rates between Blacks and Whites is the strongest predictor of the Black–White disparity in food insecurity rates since the relationships were statistically significant in both state- and county-level models. In addition, differences in poverty rates and median income between Blacks and Whites can also explain the difference in food insecurity rates between Blacks and Whites at the county level, which is consistent with the previous studies [12,13]. We found that a 1% increase in the Black unemployment rate compared with the White unemployment rate was associated with a 0.918% increase in the food insecurity rate among Blacks compared with the food insecurity rate among Whites at the state level after controlling for other variables, including difference in poverty rate, difference in homeownership rate, and difference in median income between Blacks and Whites. Moreover, a 1% increase in the unemployment rate among Blacks compared with the unemployment rate among Whites was associated with a 0.232% increase in the difference in food insecurity rate between Blacks and Whites at the county level, after controlling for the same variables. Furthermore, our results indicated that unemployment rate and median income were the strongest predictors of the food insecurity rate among Black subjects, where findings were statistically significant in both state- and county-level models. This study adds to the literature on how significantly an increase in the Black unemployment rate, a decrease in Black median income, or the wide Black–White gap among these variables can be associated with a profound rise in the food insecurity rate among Blacks and the disproportionate difference in food insecurity rates between Blacks and Whites in the US. The study shed light on important correlations between the Black–White gap in food insecurity rates and its determinants instead of suggesting causality. Future study should expand on data collection by gathering panel data of US food insecurity rates across time to examine the associations over time.

An understanding of the nature and determinants of food insecurity is imperative for improving policies that seek to reduce domestic hunger. To achieve food security, effective interventions are needed, along with adequate funding for, and increased utilization of, food and nutrition assistance programs; inclusion of nutrition education in such programs; strategies to support individual and household economic stability; and research to measure the impact on food insecurity- and health-related outcomes [9,11]. To ensure that specific populations have accessible, equitable, and eligible access to these federal nutrition programs, the research covered throughout this section indicates that further policy changes and implications are needed to ensure that Black and other disadvantaged households have equitable access to necessary nutrition programs. By ensuring further access to federal nutrition programs for low-income households, this will help improve household expenditures, health measures, social accessibility, and other determinants that are heavily impacting vulnerable families.

Furthermore, in determining the levels of food insecurity and social determinants at the state and county levels between Black and White individuals, previous research highlights how policies can negatively impact access to resources across populations. For instance, when there are economic resources available across communities, previous research notes more favorable economic conditions and job availability across communities and states. This access helps reduce economic uncertainty and increase the overall economic strength of a community [27]. Federal nutrition assistance programs, including SNAP, school breakfast and lunch programs, and summer food programs, represent a major policy commitment to meeting the food-related needs of vulnerable segments of the population. These federal programs are critically important at the state level, and the policies tied to these federal programs should be further examined to help determine the impact of these programs on food insecurity rate among Blacks or the Black–White gap in food insecurity rates.

Most importantly, to achieve food security, besides food and nutrition assistance programs or nutrition education programs, it is necessary to focus on the root causes of the Black–White gap in food insecurity rates. Strategies to support individual and household economic stability need to be enhanced. Creation of new jobs and creating more training programs for Blacks should be encouraged to reduce unemployment, increase income, and improve financial resources for Black individuals.

## Figures and Tables

**Table 1:** Descriptive statistics of Black and White characteristics at the state level (N = 51).

Variable	Mean	Std. Dev.	Min.	Max.
Black food insecurity rate	19.96	5.34	7.0	31.0
Black poverty rate	23.69	5.43	9.4	34.8
Black unemployment rate	8.94	2.00	3.8	14.1
Black homeownership rate	36.41	9.42	7.8	53.5
Black median income	42.87	9.08	30.54	69.68
White food insecurity rate	9.63	2.55	2.0	15.0
White poverty rate	9.68	2.32	5.9	16.9
White homeownership rate	71.67	5.15	50.3	80.3
White unemployment rate	4.16	0.84	2.2	6.4
White median income	69.58	15.29	47.13	141.65

Source: Map the Meal (MMG) Gap 2020 dataset. Note: Authors’ computation of summary statistics from the MMG 2020 dataset with a focus on 2019. Median income is in thousand US dollars. Number of observations is 51, including 50 states and the District of Columbia.

**Table 2:** Descriptive statistics of Black and White characteristics at the county level (N = 1675).

Variable	Mean	Std. Dev.	Min.	Max.
Black food insecurity rate	22.41	6.25	1	51
Black poverty rate	26.84	12.02	0	86.60
Black unemployment rate	9.75	6.75	0	74.60
Black homeownership rate	45.86	17.93	0	100
Black median income	39.35	18.47	2.50	250
White food insecurity rate	0.11	0.03	0.02	0.24
White poverty rate	11.95	4.63	2.70	35.60
White homeownership rate	74.81	7.34	23.70	93.60
White unemployment rate	4.73	1.73	0.30	16.50
White median income	60.25	16.85	26.07	149.09

Source: MMG 2020 dataset. Note: Authors’ computation of summary statistics from MMG 2020 dataset. The number of observations is 1675 counties.

**Table 3:** Black, White, and Black–White differential characteristics at the state level (N = 51).

Variable	Black	White	Difference
Food insecurity rate	19.96 (0.75)	9.63 (0.36)	10.33 *** (0.83)
Poverty rate	23.69 (0.76)	9.68 (0.33)	14.01 *** (0.83)
Unemployment rate	8.94 (0.28)	4.16 (0.12)	4.79 *** (0.30)
Homeownership rate	36.40 (1.32)	71.67 (0.72)	−35.26 *** (1.50)
Median income	42.87 (1.27)	69.58 (2.14)	−26.70 *** (2.49)

Source: Map the Meal (MMG) Gap 2020 dataset. Note: Authors’ computation of statistics from MMG 2020 dataset with a focus on 2019 using *t*-tests. Mean and standard errors (SE) in parentheses are reported in each cell. *** indicates statistical significance at the 1% level. All the rates are in percentage, and median income is in thousands of US dollars.

**Table 4:** Black, White, and Black–White differential characteristics at the county level (N = 1675).

Variable	Black	White	Difference
Food insecurity rate	22.41 (0.15)	11.07 (0.08)	11.34 *** (0.17)
Poverty rate	26.84 (0.29)	11.95 (0.11)	14.90 *** (0.31)
Unemployment rate	9.75 (0.16)	4.73 (0.04)	5.01 *** (0.17)
Homeownership rate	45.86 (0.44)	74.80 (0.18)	−28.94 *** (0.47)
Median income	39.35 (0.45)	60.25 (0.41)	−20.90 *** (0.61)

Source: Map the Meal (MMG) Gap 2020 dataset. Note: Authors’ computation of statistics from MMG 2020 dataset with a focus on 2019 using *t*-tests. Mean and standard errors (SE) in parentheses are reported in each cell. *** indicates statistical significance at the 1% level. All the rates are in percentage, and median income is in US dollars.

**Table 5:** Results of Model A predictors of Black–White food insecurity gap in 2019.

	(1) State Level	(2) County Level
	Dependent Variable: Black–White Food Insecurity Gap in 2019	
Difference in poverty rate	0.200	0.177 ***
	(0.187)	(0.0151)
Difference in unemployment rate	0.918 *	0.232 ***
	(0.428)	(0.0430)
Difference in homeownership rate	−0.170	0.0906 ***
	(0.0933)	(0.0101)
Difference in median income	0.0273	0.0670 ***
	(0.0669)	(0.0141)
Constant	8.397 *	3.523 ***
	(4.111)	(0.430)
N	51	1675
R^2^	0.33	0.52

Note: ***, * indicate statistical significance at the 0.1% and 5% levels, respectively (* *p* < 0.05, *** *p* < 0.001). Robust standard errors are in parentheses. Difference in food insecurity rate = Black food insecurity rate − White food insecurity rate. Difference in poverty rate = Black poverty rate − White poverty rate. Difference in unemployment rate = Black unemployment rate − White unemployment rate. Difference in homeownership rate = White homeownership rate − Black homeownership rate. Difference in median income = White median income − Black median income.

**Table 6:** Results of Model B predictors of Black food insecurity in 2019.

	(1)	(2)	(3)	(4)
	State Level		County Level	
	Dependent Variable: Black Food Insecurity 2019			
Poverty rate	−0.127		0.150 ***	
	(0.217)		(0.0221)	
Unemployment rate	0.829 *	0.794 *	0.216 ***	0.264 ***
	(0.331)	(0.323)	(0.0391)	(0.0387)
Median income	−0.291 *	−0.228 **	−0.134 ***	−0.186 ***
	(0.127)	(0.0682)	(0.0221)	(0.0202)
Homeownership rate	0.140 *	0.148 *	−0.0729 ***	−0.0893 ***
	(0.0669)	(0.0651)	(0.00786)	(0.00829)
Constant	22.94 *	17.27 ***	24.88 ***	31.24 ***
	(10.85)	(4.851)	(1.503)	(0.896)
N	51	51	1675	1675
R^2^	0.43	0.43	0.63	0.58

Note: ***, **, * indicate statistical significance at the 0.1%, 1%, and 5% levels, respectively (* *p* < 0.05, ** *p* < 0.01, *** *p* < 0.001). Robust standard errors are in parentheses.

**Figure A1:**
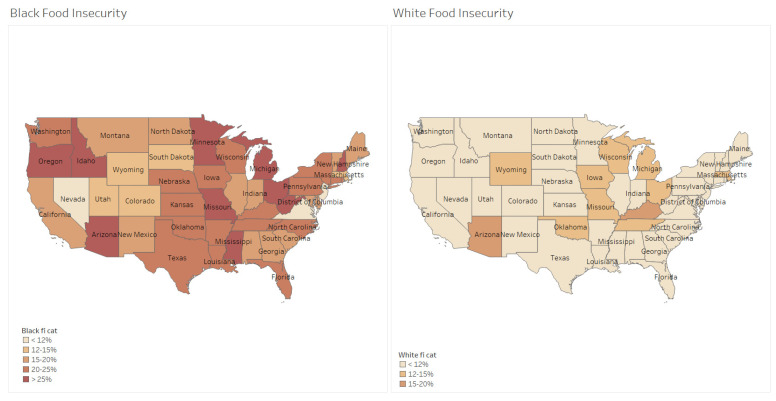
Comparison of food insecurity rates between Blacks and Whites.

**Figure A2:**
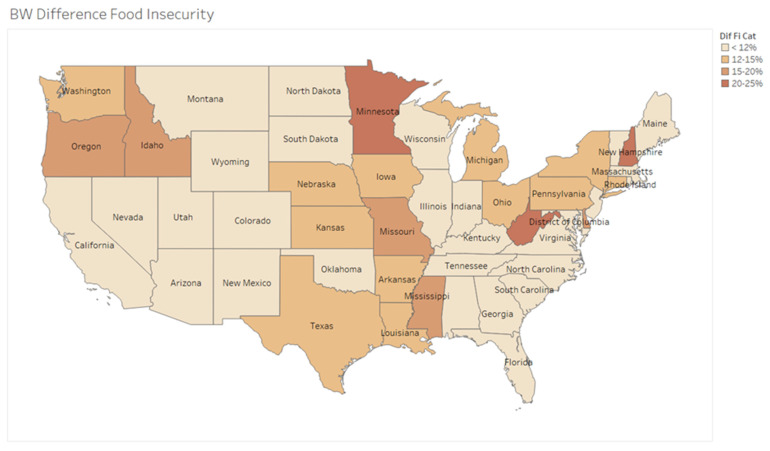
Black-White difference in food insecurity rate.

**Figure A3:**
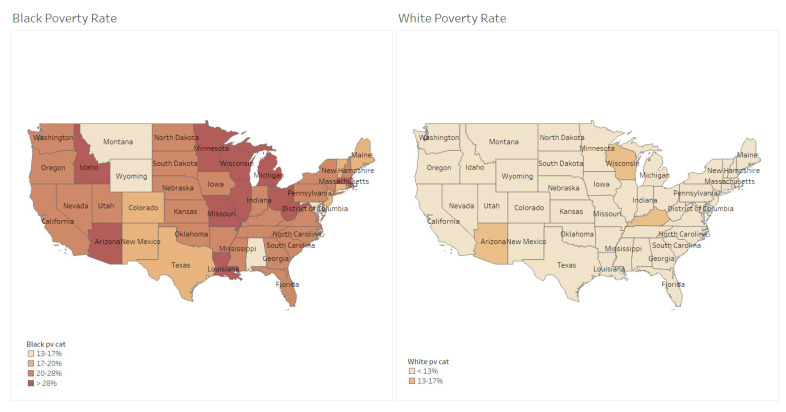
Comparison of poverty rates between Blacks and Whites.

**Figure A4:**
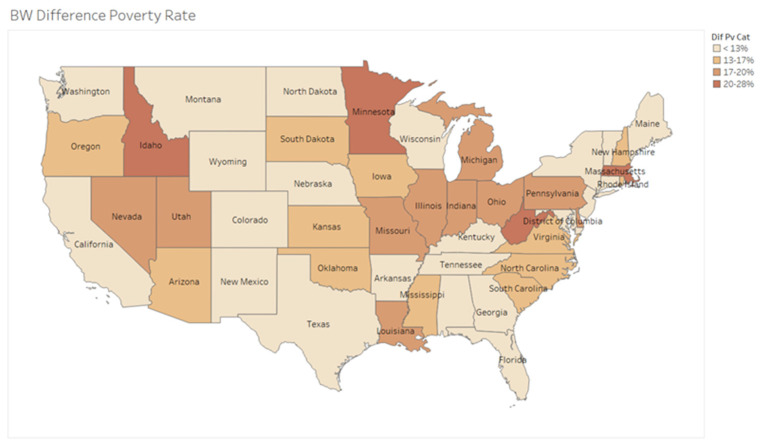
Black-White difference in poverty rate.

**Figure A5:**
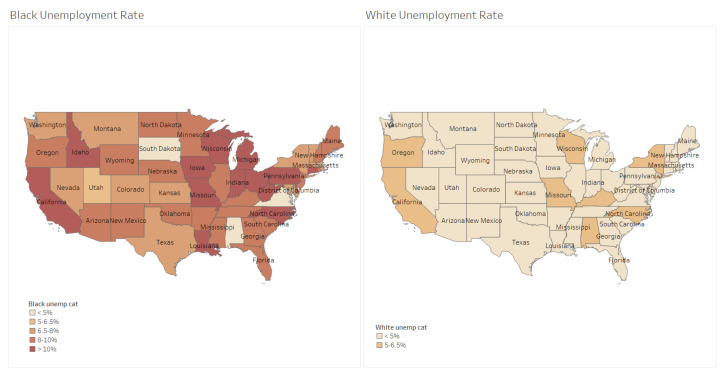
Comparison of unemployment rates between Blacks and Whites.

**Figure A6:**
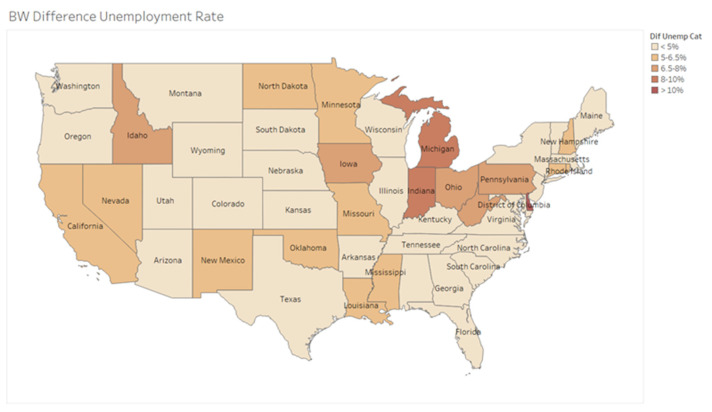
Black-White difference in unemployment rate.

**Figure A7:**
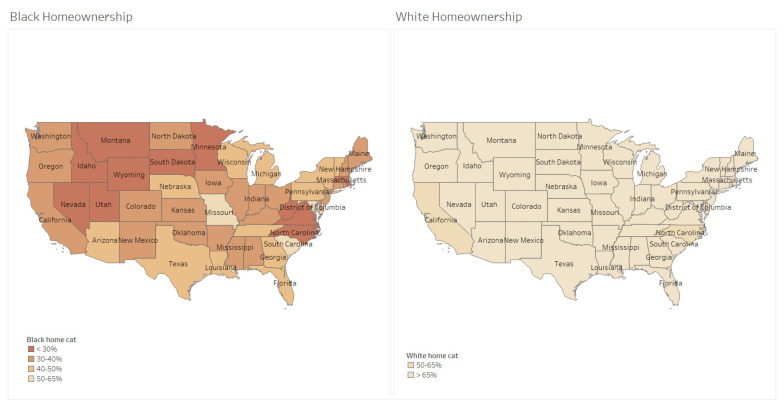
Comparison of homeownership rates between Blacks and Whites.

**Figure A8:**
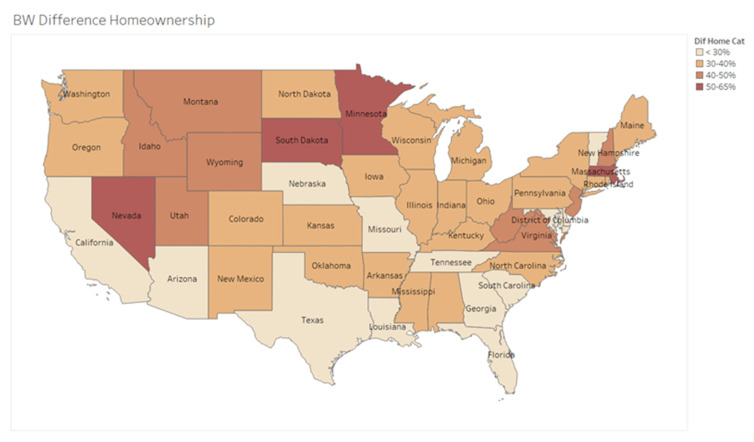
Black-White difference in homeownership rate.

**Figure A9:**
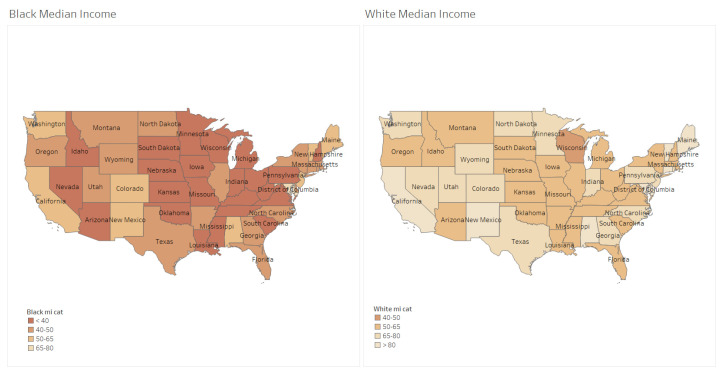
Comparison of median incomes between Blacks and Whites.

**Figure A10:**
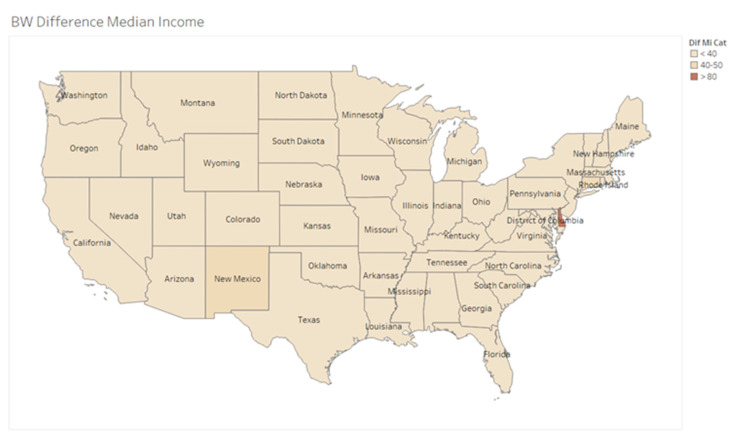
Black-White difference in median income.

**Figure A11:**
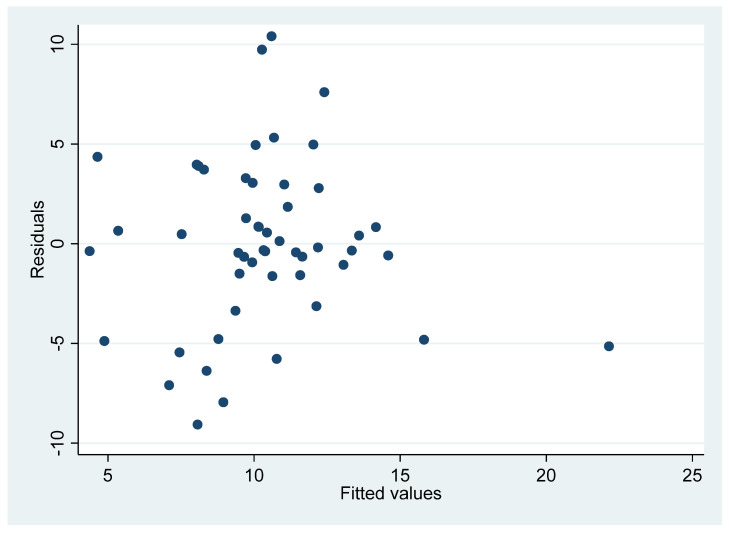
Model residual plot—no heteroscedasticity exists in the model.

## Data Availability

The data are not publicly available due to Feeding America’s data accessibility. Data sharing is available upon request.

## Appendix A

Map Visualization of the Data

## Appendix B

**Table A1:** Matrix of correlations for Model A (state level).

Variables	(1)	(2)	(3)	(4)	(5)
(1) Difference in food insecurity rate	1.00				
(2) Difference in poverty rate	0.28	1.00			
(3) Difference in unemployment rate	0.50	0.46	1.00		
(4) Difference in homeownership rate	−0.29	0.29	−0.13	1.00	
(5) Difference in median income	0.37	0.32	0.54	−0.22	1.00

**Table A2:** Matrix of correlations for Model B (county level).

Variables	(1)	(2)	(3)	(4)	(5)
(1) Difference in food insecurity rate	1.00				
(2) Difference in poverty rate	0.59	1.00			
(3) Difference in unemployment rate	0.43	0.29	1.00		
(4) Difference in homeownership rate	0.44	0.32	−0.13	1.00	
(5) Difference in median income	0.42	0.35	0.54	−0.22	1.00
